# Twelve‐month mortality outcomes for Indigenous and non‐Indigenous people admitted to intensive care units in Australia: a registry‐based data linkage study

**DOI:** 10.5694/mja2.51763

**Published:** 2022-10-30

**Authors:** Paul J Secombe, Alex Brown, Michael J Bailey, Sue Huckson, Shaila Chavan, Edward Litton, David Pilcher

**Affiliations:** ^1^ Alice Springs Hospital Alice Springs NT; ^2^ Monash University Melbourne VIC; ^3^ Australian National University Canberra ACT; ^4^ South Australian Health and Medical Research Institute Adelaide SA; ^5^ Centre for Outcome and Resource Evaluation (CORE) Australian and New Zealand Intensive Care Society (ANZICS) Melbourne VIC; ^6^ Fiona Stanley Hospital Perth WA; ^7^ The Alfred Hospital Melbourne VIC

## Abstract

**Objective:**

To compare longer term (12‐month) mortality outcomes for Indigenous and non‐Indigenous people admitted to intensive care units (ICUs) in Australia.

**Design, setting, participants:**

Retrospective registry‐based data linkage cohort study; analysis of all admissions of adults (16 years or older) to Australian ICUs, 1 January 2017 – 31 December 2019, as recorded in the Australian and New Zealand Intensive Care Society (ANZICS) Adult Patient Database (APD), linked using the SLK‐581 key to National Death Index data.

**Main outcome measures:**

Unadjusted and adjusted mortality risk, censored at twelve months from the start of index ICU admission. Secondary outcomes were unadjusted and adjusted mortality twelve months from admission to the ICU.

**Results:**

The APD recorded 330 712 eligible ICU admissions during 2017–2019 (65% of all ICU admissions registered), of which 11 322 were of Indigenous people (3.4%). Median age at admission was lower for Indigenous patients (51.2 [IQR, 36.7–63.6] years) than for non‐Indigenous patients (66.5 [IQR, 52.7–76.1] years). Unadjusted mortality risk was similar for Indigenous and non‐Indigenous patients (hazard ratio, 1.01; 95% CI, 0.97–1.06), but was higher for Indigenous patients after adjusting for age, admission diagnosis, illness severity, hospital type, jurisdiction, remoteness and socio‐economic status (adjusted hazard ratio, 1.20; 95% CI, 1.14–1.27). Twelve‐month mortality was higher for Indigenous than non‐Indigenous patients (adjusted odds ratio, 1.24; 95% CI, 1.16–1.33).

**Conclusions:**

Twelve‐month mortality outcomes are poorer for people admitted to ICUs in Australia than for the general population. Further, after adjusting for age and other factors, survival outcomes are poorer for Indigenous than non‐Indigenous people admitted to ICUs. Critical illness may therefore contribute to shorter life expectancy among Indigenous Australians.

**The known:** Hospital and ICU admission rates are about 1.3 times as high for Indigenous people as for other Australians, but ICU and hospital mortality are similar for both groups.

**The new:** In our analysis of people admitted to ICUs during 2017–2019, risk of death and 12‐month mortality among those discharged from the ICU alive were higher for Indigenous than non‐Indigenous people after adjusting for age and other baseline differences.

**The implications:** Hospital mortality does not include the full mortality burden of critical illness. After taking the lower median age of Indigenous ICU patients into account, their mortality outcomes are significantly poorer than for non‐Indigenous patients.

Rates of ill‐health are higher and life expectancy lower for indigenous peoples than for other residents of many countries.^1^ Further, critical illness is associated with significant morbidity, and mortality following intensive care unit (ICU) care is higher than the general population death rate.^2^, ^3^, ^4^, ^5^, ^6^, ^7^, ^8^, ^9^ However, few investigations of longer term outcomes have been published, particularly for critically ill Aboriginal and Torres Strait Islander (Indigenous) Australians.^6^, ^8^

Hospital and ICU admission rates are about 1.3 times as high for Indigenous people as for other Australians, but ICU and hospital mortality outcomes are similar.^8^, ^10^, ^11^ The burden of chronic disease is higher for Indigenous Australians and the median age at onset lower, leading to reduced life expectancy compared with other Australians.^12^, ^13^, ^14^ Although smaller studies have found that mortality directly attributable to critical illness does not contribute to the mortality difference, long term national data are not available.^6^, ^8^ We therefore undertook a registry‐based data linkage study to compare longer term (12‐month) mortality outcomes for Indigenous and non‐Indigenous Australians admitted to ICUs.

## Methods

We conducted a retrospective registry‐based data linkage cohort study of people admitted to Australian ICUs during 1 January 2017 – 31 December 2019. The Australian and New Zealand Intensive Care Society (ANZICS) Adult Patient Database (APD), one of four clinical quality registries managed by the ANZICS Centre for Outcome and Resource Evaluation (CORE), was linked to the National Death Index (NDI) using a statistical linkage key (SLK‐581)^15^ introduced to the APD in 2017. The Australian Institute of Health and Welfare data linkage unit undertook the linkage and provided the linked dataset to ANZICS CORE.

### Data extraction and definitions

We identified all ICU admissions of adults resident in Australia (16 years or older) to one of the 168 Australian ICUs that contribute data to the ANZICS APD and use the SLK‐581. The APD included data for more than 90% of ICU admissions during 2017–2019. Recording of Indigenous status has been mandatory in the APD since 2010; in the ANZICS database, “Indigenous” includes all Aboriginal and Torres Strait Islander Australians.^16^ Indigenous status for the NDI is determined from the death notification form and the cause of death certificate completed by the certifying medical officer.^17^ If the information in the two datasets was discordant, we preferred APD coding of Indigenous status. We excluded ICU admissions when they were for palliative care or organ donation, the ICU or hospital outcome was unknown, or hospital discharge date or Indigenous status were not recorded in the APD. In cases of multiple ICU admission, only the index episode of care was included, and inter‐hospital transfers were excluded at the level of the receiving hospital. We also excluded episodes when a clear data mismatch was evident (eg, NDI date of death was earlier than that of ICU admission).

We extracted data for demographic characteristics (age, sex, residential postcode), comorbid conditions (defined by the ANZICS APD data dictionary^16^), and the need for mechanical ventilation. Illness severity was based on Acute Physiology and Chronic Health Evaluation (APACHE) III^18^ and Australian and New Zealand Intensive Care Risk of Death (ANZROD) scores; ANZROD scores are derived from selected APACHE III components and data for other variables collected in Australia and New Zealand.^19^ Postcode‐based remoteness and socio‐economic status were based on the 2011 Accessibility and Remoteness Index of Australia (ARIA+)^20^ and the Socio‐Economic Indexes for Areas (SEIFA) 2016 Index of Relative Socio‐Economic Advantage and Disadvantage (IRSAD).^21^ We included these two variables to account for the larger proportions of Indigenous Australians living in outer regional, remote, and very remote areas and in areas of lower socio‐economic status, where life expectancy is shorter for both Indigenous and non‐Indigenous Australians.^11^, ^22^

Reason for ICU admission was based on the ANZICS modification of the APACHE III/IV diagnostic coding system.^16^ Individual admission diagnoses were grouped into eight major system‐based categories. Date of death was obtained from the NDI, which comprises person‐level records for all deaths in Australia, supplied by jurisdictional registrars of Births, Deaths, and Marriages.

### Outcomes

The primary outcomes were unadjusted and adjusted mortality risk, censored at twelve months from the start of the index ICU admission. Secondary outcomes were unadjusted and adjusted in‐hospital mortality and for mortality 30 days, 60 days, and 12 months after ICU admission.

### Statistical analysis

Analyses were undertaken in Stata 15.1. We summarise data as counts and proportions, means with standard deviations (SDs), or medians with interquartile ranges (IQRs); the statistical significance of between‐group differences was assessed in χ^2^ or Student *t* tests (normally distributed data) or Wilcoxon rank‐sum tests (non‐normally distributed data).

Survival time is depicted in Kaplan–Meier curves, and between‐group differences were assessed in log‐rank tests. Longer term mortality was referenced to Australian Bureau of Statistics age‐ and sex‐matched life tables for the non‐Indigenous and Indigenous populations (5‐year blocks).^23^ Analyses stratified by age are reported in similar fashion.

We compared mortality risk for Indigenous and non‐Indigenous patients in a multivariable Cox proportional hazard model, adjusted for covariates selected on the basis of baseline differences between Indigenous and non‐Indigenous patients and for other covariates that influence mortality (illness severity, hospital type, jurisdiction, socio‐economic status, remoteness, admission diagnosis).^2^, ^4^, ^8^, ^11^ Given the difference in median age between Indigenous and non‐Indigenous patients on ICU admission, illness severity was adjusted only for the physiological and chronic health components of APACHE III, and age was included as a separate continuous variable.^11^ We report hazard ratios (HRs) with 95% confidence intervals (CIs). The assumption of proportional hazards was assessed visually in log‐log graphs of survival time.

We assessed 12‐month mortality in a mixed effects multivariable logistic regression model similarly adjusted for potential confounders, with patients clustered by site, and site treated as a random effect.^2^, ^4^, ^8^, ^11^ We report odds ratios (ORs) with 95% CIs. The discrimination of the multivariable model was assessed by calculating the area under the receiver operating characteristic (AUROC) curve.

In supplementary analyses, we quantified the concordance of APD and NDI classification of inpatient mortality and Indigenous status with Krippendorff's alpha coefficient. As there is no gold standard for defining Indigenous status in health records,^24^ we assessed the sensitivity of our findings to the APD definition by repeating our analyses using a broader definition (Indigenous status recorded in either database).

### Ethics approval

Our study was approved by the Central Australian Human Research Ethics Committee (CA‐21‐3965), and our report conforms with the Strengthening the Reporting of Observational studies in Epidemiology (STROBE) statement (Supporting Information, table 1).^25^

## Results

The APD recorded 508 449 ICU admissions during 2017–2019; we included 330 712 admissions in our analyses (65%), of which 11 322 were of Indigenous people (3.4%) (Box 1). Median age at admission was lower for Indigenous (51.2 years; IQR, 36.7–63.6 years) than non‐Indigenous patients (66.5 years; IQR, 52.7–76.1 years). Two‐thirds of Indigenous patients (7698 people, 68%) were admitted to ICUs in New South Wales, Western Australia, or Queensland, consistent with the geographic distribution of Indigenous Australians;^26^ the proportion of Indigenous ICU admissions was largest in the Northern Territory (1829 of 3547, 51.6%). Among patients for whom residential postcodes could be mapped to ARIA+ categories, 5773 of 11 076 Indigenous people (52.1%) and 44 561 of 315 170 non‐Indigenous people (14.1%) lived in outer regional, remote, or very remote areas (Supporting Information, table 2). The median IRSAD score was lower for Indigenous (951; IQR, 897–991) than non‐Indigenous patients (991; IQR, 945–1051) (Supporting Information, table 2; distribution: Box 2).

Box 1 **Selection of linked Australian and New Zealand Intensive Care Society Centre for Outcome and Resource Evaluation Adult Patient Database (ANZICS CORE APD) intensive care unit (ICU) admissions and National Death Index (NDI) records for our analysis**

**Figure:**
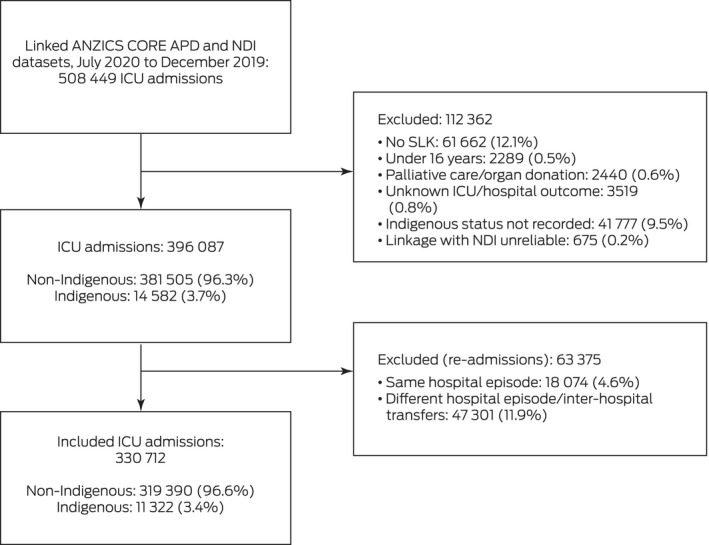
SLK = statistical linkage key.

Box 2 Area‐level Index of Relative Socio‐economic Advantage and Disadvantage (IRSAD) of 327 031 people* admitted to intensive care in Australia, 2017–2019, by Indigenous status and decile

**Figure:**
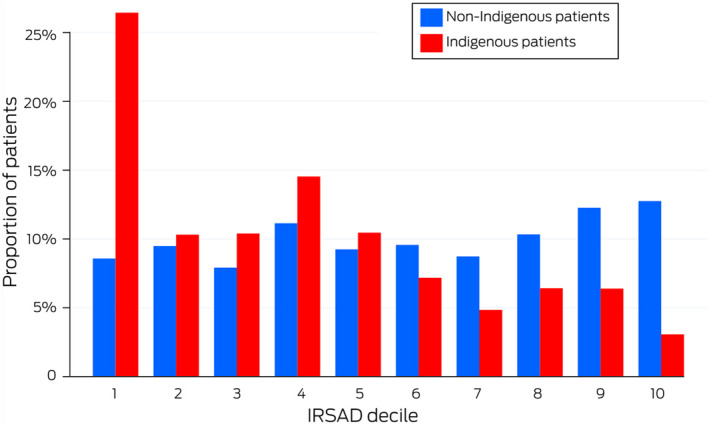
* 315 866 non‐Indigenous, 11 165 Indigenous patients for whom residential postcode could be mapped to IRSAD decile. The raw data for this figure are included in Supporting Information, table 3.

### Intensive care unit admission characteristics

The proportion of planned (elective) ICU admissions was smaller for Indigenous than non‐Indigenous patients (21.6% *v* 41.1%). Larger proportions of Indigenous patients had chronic (dialysis‐dependent) renal failure (8.2% *v* 2.9%) or chronic liver dysfunction (2.8% *v* 1.5%). The prevalence of multimorbidity was similar for Indigenous and non‐Indigenous people (5.4% *v* 5.0%), despite the difference in median age (Box 3).

Box 3 **Admission characteristics, comorbid chronic illnesses, and illness severity at admission for 330 712 people admitted to intensive care units (ICUs) in Australia, 2017–2019, by Indigenous status**

**Table:** 

Characteristic	All patients	Non‐Indigenous patients	Indigenous patients
Patients	330 712	319 390	11 322
Planned (elective) admissions*	133 768 (40.4%)	131 325 (41.1%)	2443 (21.6%)
Admission with ICU level of care^†^	105 164 (31.8%)	101 317 (31.7%)	3847 (34.0%)
Length of stay (days), median (IQR)			
ICU	1.7 (0.9–3.1)	1.7 (0.9–3.0)	1.9 (1.0–3.7)
Hospital	7.8 (4.2–13.9)	7.8 (4.2–13.9)	7.2 (3.7–13.9)
Mechanical ventilation	105 793 (32.0%)	101 231 (31.7%)	4562 (40.3%)
Chronic diseases*			
Cardiovascular	27 251 (8.2%)	26 350 (8.3%)	901 (8.0%)
Respiratory	24 348 (7.4%)	23 446 (7.3%)	902 (8.0%)
Immunosuppressed^‡^	21 238 (6.4%)	20 749 (6.5%)	489 (4.3%)
Metastatic disease	12 901 (3.9%)	12 680 (4.0%)	221 (2.0%)
Renal	10 157 (3.1%)	9223 (2.9%)	934 (8.2%)
Liver	4986 (1.5%)	4669 (1.5%)	317 (2.8%)
Leukaemia	3467 (1.1%)	3412 (1.1%)	55 (0.5%)
Lymphoma	2834 (0.9%)	2784 (0.9%)	50 (0.4%)
Two or more chronic diseases	16 421 (5.0%)	15 807 (5.0%)	614 (5.4%)
APACHE III, median score (IQR)	46 (34–62)	46 (34–61)	45 (32–64)
Age score	13 (5–17)	13 (5–17)	5 (0–11)
Acute physiology score	33 (24–47)	33 (24–47)	38 (26–55)
Risk of death, median (IQR)			
APACHE III	4.3% (1.6–13.0%)	4.3% (1.6–12.9%)	4.6% (1.5–14.4%)
ANZROD	1.3% (0.4–5.4%)	1.2% (0.4–5.4%)	1.6% (0.5–6.3%)

ANZROD = Australian and New Zealand Risk of Death; APACHE III = Acute Physiology and Chronic Health Evaluation III; IQR = interquartile range.

* As defined by the Australian and New Zealand Intensive Care Society Centre for Outcome and Resource Evaluation Adult Patient Database (ANZICS CORE APD) data dictionary.^16^

† As defined by the ANZICS CORE APD data dictionary: admissions of patients requiring one of the following: “invasive ventilation; non‐invasive ventilation (> 50% of stay or continuously > 6 h); 1:1 nursing; continuous renal replacement therapy.” Admissions of patients requiring the specific expertise of the ICU environment but not fitting this definition are defined as high dependency unit admissions.^16^

‡ ANZICS‐modified APACHE III chronic disease definition: immunosuppressed, immunosuppressive therapy, immune‐suppressing disease, acquired immune deficiency syndrome.^16^

The proportions of people admitted because of sepsis (11.6% *v* 7.2%), respiratory illness (16.6% *v* 14.7%), or trauma (7.6% *v* 4.6%) were larger for Indigenous than non‐Indigenous patients (Box 4). The proportion who were mechanically ventilated was larger for Indigenous patients (40.3% *v* 31.7%), and the median APACHE III illness severity scores were similar, but the median age score was lower for Indigenous patients (Box 3).

Box 4 Admission diagnosis categories for 330 712 people admitted to intensive care units (ICUs) in Australia, 2017–2019, by Indigenous status*

**Figure:**
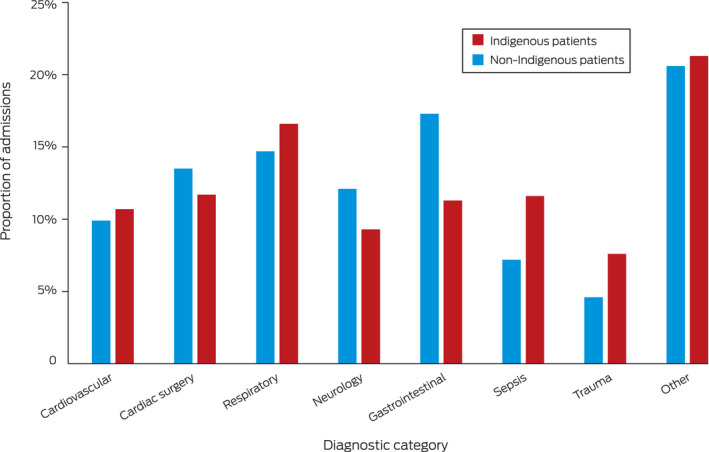
* Data missing for 24 Indigenous and 625 non‐Indigenous patients. The raw data for this figure are included in Supporting Information, table 4.

### Primary and secondary outcomes

Median time to death was similar for the 1793 Indigenous (22 days; IQR, 4–128 days) and the 50 108 non‐Indigenous patients (26 days; IQR, 5–137 days) who died within twelve months of ICU admission. Unadjusted mortality risk was similar for Indigenous and non‐Indigenous patients (HR, 1.01; 95% CI, 0.97–1.06) (Box 5, Box 6); after adjusting for age, admission diagnosis, illness severity (APACHE III, with age score removed), hospital type, jurisdiction, remoteness and socio‐economic status, mortality risk was greater for Indigenous patients (adjusted HR, 1.20; 95% CI, 1.14–1.27) (Box 5). Unadjusted mortality risk was also higher for Indigenous than non‐Indigenous patients aged 50 years or younger (HR, 1.51; 95% CI, 1.38–1.66) and for those over 50 years of age (HR, 1.21; 95% CI, 1.14–1.28) (Box 7).

Box 5 **Summary of primary and secondary outcomes for 330 712 people admitted to intensive care units (ICUs) in Australia, 2017–2019, by Indigenous status**

**Table:** 

Characteristic	All patients	Non‐Indigenous patients	Indigenous patients
**Primary outcome: risk of death**			
All patients	330 712	319 390	11 322
Deaths (censored at 12 months)	51 901	50 108	1793
Risk of death			
Hazard ratio (95% CI)	—	1	1.01 (0.97–1.06)
Adjusted hazard ratio (95% CI)*	—	1	1.20 (1.14–1.27)
Time from ICU admission to death (days), median (IQR)^†^	26 (5–137)	26 (5–137)	22 (4–128)
**Secondary outcomes: deaths**			
In‐hospital mortality	24 449 (7.4%)	23 573 (7.4%)	876 (7.7%)
Odds ratio (95% CI)	—	1	1.05 (0.98–1.13)
Adjusted odds ratio (95% CI)*	—	1	1.11 (1.01–1.22)
30‐day mortality	27 141 (8.2%)	26 167 (8.2%)	974 (8.6%)
Odds ratio (95% CI)	—	1	1.05 (0.99–1.13)
Adjusted odds ratio (95% CI)*	—	1	1.11 (1.01–1.21)
90‐day mortality	35 171 (10.6%)	33 933 (10.6%)	1238 (10.9%)
Odds ratio (95% CI)	—	1	1.03 (0.92–1.09)
Adjusted odds ratio (95% CI)*	—	1	1.18 (1.09‐1.28)
12‐month mortality	51 901 (15.7%)	50 108 (15.7%)	1793 (15.8%)
Odds ratio (95% CI)	—	1	1.01 (0.96–1.06)
Adjusted odds ratio (95% CI)*	—	1	1.24 (1.16–1.33)
Time from hospital discharge to death (days), median (IQR)^‡^	125 (51–233)	126 (51–233)	120 (49–229)

CI = confidence interval; IQR = interquartile range.

* Adjusted for age, admission diagnosis, illness severity (APACHE III, with age score removed), and socio‐economic status. For further details, see Supporting Information, table 5.

† For patients who died within twelve months of ICU admission.

‡ For deaths among the 306 263 patients (295 817 non‐Indigenous, 10 446 Indigenous patients) who were discharged alive from hospital but died within twelve months of ICU admission.

Box 6 **Kaplan–Meier survival curves (with 95% confidence intervals) for people admitted to intensive care units (ICUs), Australia, 2017–2019, by Indigenous status (censored at twelve months)**

**Figure:**
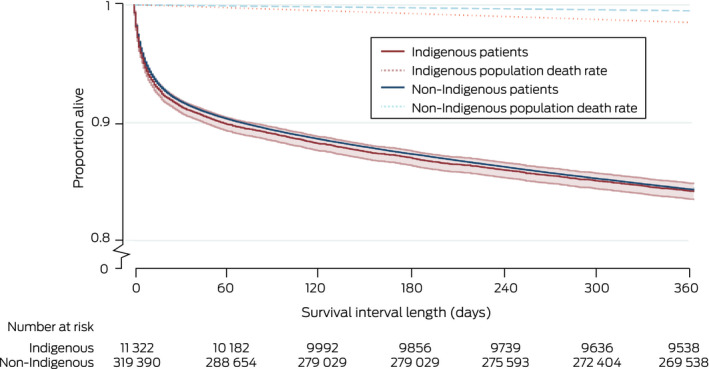
Indigenous *v* non‐Indigenous patients: *P* = 0.60 (log‐rank test).

Box 7 **Kaplan–Meier survival curves (with 95% confidence intervals) for people admitted to intensive care units (ICUs), Australia, 2017–2019, by Indigenous status and age group (censored at twelve months)**

**Figure:**
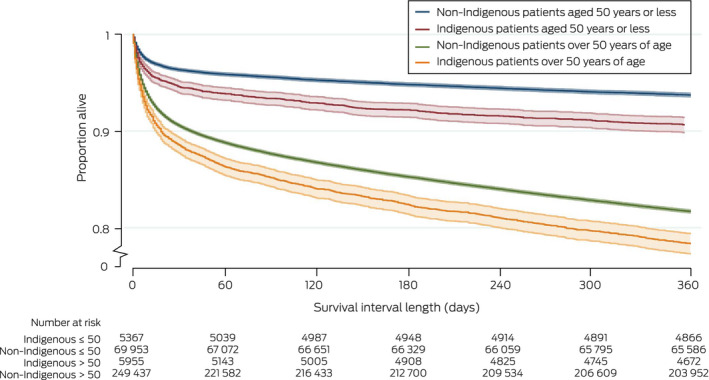
For each age group: Indigenous *v* non‐Indigenous patients: *P* < 0.001 (log‐rank test).

Unadjusted 12‐month mortality was similar for Indigenous and non‐Indigenous patients discharged from the ICU alive (15.8% *v* 15.7%; OR, 1.01; 95% CI, 0.96–1.06), as were other mortality outcomes (in‐hospital, 30‐day, 90‐day mortality). After adjusting for age, diagnosis, illness severity, and socio‐economic status, 12‐month mortality was higher for Indigenous than non‐Indigenous patients (adjusted OR, 1.24; 95% CI, 1.16–1.33) (Box 5). Twelve‐month mortality exceeded mean age‐, sex‐, and Indigenous status‐matched population death rates (Indigenous, 1.5%; non‐Indigenous, 0.5%) for both Indigenous and non‐Indigenous people (Box 6).

### Supplementary analyses

The concordance between APD hospital deaths and the NDI data on inpatient mortality was high (Krippendorff's alpha = 0.97; Supporting Information, table 6), but not for the Indigenous status of those who died within twelve months of admission (Krippendorff's alpha = 0.55, Supporting Information, table 7). A sensitivity analysis in which people were classified as Indigenous when this status was recorded in either dataset added 353 Indigenous patients, but the baseline characteristics of the two groups were similar to those in the main analysis (Supporting Information, table 8). Mortality risk (adjusted HR, 1.45; 95% CI, 1.38–1.52) and 12‐month mortality (17.8% *v* 15.0%; adjusted OR, 1.55; 95% CI, 1.46–1.65) were each less favourable for Indigenous patients (Supporting Information, table 9).

A larger proportion of patients without SLK‐581s were admitted to tertiary ICUs than of those with keys (45.7% *v* 38.8%), smaller proportions were admitted to private ICUs (21.2% *v* 32.9%), and their median APACHE III score was higher (47; IQR, 34–61 *v* 46; IQR, 34–62). However, no clinically meaningful differences between linked and unlinked data were identified (Supporting Information, table 10).

## Discussion

Unadjusted survival time and 12‐month mortality were similar for Indigenous and non‐Indigenous Australians admitted to ICUs during 2017–2019. However, the median age at ICU admission was 15 years lower for Indigenous patients. After adjusting for age and other factors, risk of death in the twelve months following ICU admission was greater for Indigenous patients, and 12‐month mortality higher.

In‐hospital mortality for Indigenous and non‐Indigenous Australian ICU patients is similar.^11^, ^14^ Our findings suggest that critical illness may nevertheless contribute to earlier death among Indigenous Australians, and consequently to lower life expectancy. It is likely that the difference is less directly attributable to the ICU admission than to a complex interplay between pre‐ and post‐hospitalisation factors, socio‐economic disadvantage, remoteness, and chronic disease trajectory. Primary and public health interventions for preventing and managing chronic disease could reduce the incidence of early death.

Apart from a study of outcomes for non‐elective ICU admissions in South Australia,^8^ information on long term outcomes for critically ill Indigenous patients is limited. In our study, we included data for both elective and non‐elective ICU admissions and from all Australian states and territories, but our findings (both unadjusted and adjusted outcomes) and those of the earlier study were similar.

An often overlooked problem when comparing long term outcomes is distinguishing between studies that set the onset of critical illness as the baseline and those that examine longer term outcomes for people who survive to hospital discharge.^2^, ^4^, ^7^ We recruited patients on admission to obtain a clearer picture of outcomes for all critically ill people admitted to ICUs. Nevertheless, overall 12‐month mortality was similar to that reported in other settings during the past decade.^3^, ^5^, ^7^ This may reflect advances in the post‐hospital care of people with chronic disease, or the universal availability of publicly funded health care in Australia.

The higher adjusted mortality among Indigenous than non‐Indigenous patients twelve months after admission for ICU care is disturbing, and why the median age of Indigenous people who require ICU care is 15 years lower than for non‐Indigenous patients is unclear. Possible explanations include more rapid disease progression and restricted access to pre‐ and post‐ICU care in public and primary health care. Each possibility warrants further investigation. It was recently reported, for example, that intensive post‐ICU management improves outcomes for people successfully treated for sepsis.^27^

### Limitations

The major strength of our population‐based cohort linkage study was the robustness of the two databases used. The high degree of inpatient death data concordance indicates high quality of data collection for variables other than Indigenous status.

However, the relatively recent introduction of the SLK‐581 meant that we could match APD data to NDI data only from 2017, although this also means that our findings reflect contemporary ICU treatment and management of chronic conditions. Second, we could not assess pre‐hospital factors (eg, functional status), living arrangements, or health care access and use. Third, database analyses are limited by the variables included in the databases. Neither the APD nor the NDI include patient‐reported outcomes, such as quality of life, or data on risk factors for health service use, including availability of family and other social support. Fourth, we could not account for patients lost to follow‐up because of migration. Fifth, we excluded 35% of ICU admissions in the APD database, including 9.5% for which the Indigenous status of the patient was not recorded (Box 1). Sixth, as we included data only for index admissions, we may have underestimated mortality among patients transferred to other ICUs; however, the degree of concordance between the APD and NDI for inpatient deaths means the extent of this problem was probably minor. Seventh, the scores we used to adjust for illness severity were calibrated using broad datasets, not for specific subgroups, such as Indigenous people.

Finally, we found that concordance between the APD and NDI regarding Indigenous status was poor. A decade has passed since the publication of the national best practice guidelines for Indigenous status in health datasets^17^ and inclusion of Indigenous status in the admitted patient care national minimum dataset.^28^ Not all Indigenous patients may be identified as such in health care data,^24^ but our sensitivity analysis, applying an expanded definition of Indigenous status, yielded similar results to the main analysis.

### Conclusion

Although in‐hospital mortality is similar for Indigenous and non‐Indigenous ICU patients, longer term risk of death and 12‐month mortality are each higher for Indigenous than non‐Indigenous people. Further, as the median age of Indigenous patients is about 15 years lower than for non‐Indigenous ICU patients, the differences for critically ill Indigenous people are even more marked after adjusting for age and other factors.

## Open access

Open access publishing facilitated by Monash University, as part of the Wiley – Monash University agreement via the Council of Australian University Librarians.

## Competing interests

No relevant disclosures.

## Data sharing statement

A full statement is included at the end of the Supporting Information.

## Supporting information




Table S1.
