# Supplementary material for: Twelve‐month mortality outcomes for Indigenous and non‐Indigenous people admitted to intensive care units in Australia: a registry‐based data linkage study
Source: Med J Aust. 2022 Oct 30;218(2):77–83. doi: 10.5694/mja2.51763 (PMC10092659; doi:10.5694/mja2.51763)
Supplement: Supplementary file 1 — Table S1. [file MJA2-218-77-s001.pdf]

MJA
The Medical Journal of Australia

Supporting Information

Supplementary results

**This appendix was part of the submitted manuscript and has been peer reviewed.
It is posted as supplied by the authors.**

Appendix to: Secombe PJ, Brown A, Bailey MJ, et al. Twelve-month mortality outcomes for Indigenous and non-Indigenous people admitted to intensive care units in Australia: a registry-based data linkage study. *Med J Aust* 2022; doi: 10.5694/mja2.51763.

Table 1. STROBE statement: checklist of items that should be included in reports of cohort studies[1]

	Recommendation	Response
**Title & abstract**	(*a*) Indicate the study's design with a commonly used term in the title or the abstract	Long Term Outcomes of Indigenous Survivors of Critical Illness: A Registry-Based Data-Linkage Study
	(*b*) Provide in the abstract an informative and balanced summary of what was done and what was found	Presented in standardised format
**Introduction**		
Background/rationale	Explain the scientific background and rationale for the investigation being reported	Previous studies have demonstrated hospital mortality equivalence, but there is no data for long term outcomes.
Objectives	State specific objectives, including any prespecified hypotheses	To examine the long-term outcomes of critically ill adult Indigenous patients compared to non-Indigenous patients.
**Methods**		
Study design	Present key elements of study design early in the paper	Retrospective registry-based data-linkage cohort study
Setting	Describe the setting, locations, and relevant dates, including periods of recruitment, exposure, follow-up, and data collection	Australia<br>1 Jan 2017 – 31 Dec 2019<br>Data collection tool specified (COMET) and National Death Index
Participants	(*a*) Give the eligibility criteria, and the sources and methods of selection of participants. Describe methods of follow-up	Exclusions: no outcome data<br>ICU re-admission<br>Age < 16 years<br>Missing Indigenous status<br>Failed NDI linkage<br>Admitted for purposes of organ donation
	(*b*) For matched studies, give matching criteria and number of exposed and unexposed	N/A
Variables	Clearly define all outcomes, exposures, predictors, potential confounders, and effect modifiers. Give diagnostic criteria, if applicable	ANZICS APD Data Dictionary[4]
Data sources/ measurement	For each variable of interest, give sources of data and details of methods of assessment (measurement). Describe comparability of assessment methods if there is more than one group	All data held within the ANZICS CORE APD
Bias	Describe any efforts to address potential sources of bias	
Study size	Explain how the study size was arrived at	N/A convenience sampling
Quantitative variables	Explain how quantitative variables were handled in the analyses. If applicable, describe which groupings were chosen and why	
Statistical methods	(*a*) Describe all statistical methods, including those used to control for confounding	Statistical Methods section in Methods
	(*b*) Describe any methods used to examine subgroups and interactions	
	(*c*) Explain how missing data were addressed	Mostly excluded
	(*d*) If applicable, explain how loss to follow-up was addressed	No loss to followup
	(*e*) Describe any sensitivity analyses	Hierarchical regression<br>Comparison of linked and unlinked episodes care<br>Concordance between datasets for Indigenous status and inpatient mortality
**Results**		
Participants	(a) Report numbers of individuals at each stage of study—eg numbers potentially eligible, examined for eligibility, confirmed eligible, included in the study, completing follow-up, and analysed	Box 1
	(b) Give reasons for non-participation at each stage	Box 1
	(c) Consider use of a flow diagram	Box 1

	Recommendation	Response
Descriptive data	(a) Give characteristics of study participants (eg demographic, clinical, social) and information on exposures and potential confounders	Supporting information table 2
	(b) Indicate number of participants with missing data for each variable of interest	N/A
	(c) Summarise follow-up time (eg, average and total amount)	Mortality censored at 365 days<br>Minimum follow-up 1 day<br>Maximum follow up 1437
Outcome data	Report numbers of outcome events or summary measures over time	Table 3
Main results	(*a*) Give unadjusted estimates and, if applicable, confounder-adjusted estimates and their precision (eg, 95% confidence interval). Make clear which confounders were adjusted for and why they were included	Box 5<br>Supporting information tables 4, 7 – 9.
	(*b*) Report category boundaries when continuous variables were categorized	N/A
	(*c*) If relevant, consider translating estimates of relative risk into absolute risk for a meaningful time period	Presented as hazard ratio for mortality risk and odds ratio as appropriate
Other analyses	Report other analyses done—eg analyses of subgroups and interactions, and sensitivity analyses	Supporting information, tables 7 - 9
**Discussion**		
Key results	Summarise key results with reference to study objectives	Paragraph 1 and 2
Limitations	Discuss limitations of the study, taking into account sources of potential bias or imprecision. Discuss both direction and magnitude of any potential bias	Limitations section
Interpretation	Give a cautious overall interpretation of results considering objectives, limitations, multiplicity of analyses, results from similar studies, and other relevant evidence	Although in-hospital mortality is similar for Indigenous and non-Indigenous ICU patients, longer term risk of death and 12-month mortality are each higher for Indigenous than non-Indigenous people. Further, as the median age of Indigenous patients is about 15 years lower than for non-Indigenous ICU patients, the differences for critically ill Indigenous people are even more marked after adjusting for age and other factors
Generalisability	Discuss the generalisability (external validity) of the study results	Limitations section
**Other information**		
Funding	Give the source of funding and the role of the funders for the present study and, if applicable, for the original study on which the present article is based	Acknowledgment section

* Give information separately for exposed and unexposed groups.

Table 2. Characteristics of 330 712 people admitted to intensive care units (ICUs) in Australia, 2017–2019, by Indigenous status

Characteristic	All patients	Non-Indigenous patients	Indigenous patients
Number of people	330 712	319 390	11 322
Age at admission (years), median (IQR)	66.1 (51.9–75.8)	66.5 (52.7–76.1)	51.2 (36.7–63.6)
50 years or younger	75 320 (22.8%)	69 953 (21.9%)	5367 (47.4%)
Over 50 years	255 392 (77.2%)	249 437 (78.1%)	5955 (52.6%)
Sex			
Men	185 522 (56.1%)	179 705 (56.3%)	5817 (51.4%)
Women	144 915 (43.8%)	139 415 (43.7%)	5500 (48.9%)
Other	189 (0.1%)	184 (0.1%)	5 (< 0.1%)
Missing data	86 (< 0.1%)	86 (< 0.1%)	0
State (ICU admission)			
New South Wales	109 878 (33.2%)	106 346 [96.8%]	3532 [3.2%]
Victoria	84 705 (25.6%)	83 926 [99.1%]	779 [0.9%]
Queensland	64 910 (19.6%)	62 777 [96.7%]	2133 [3.3%]
South Australia	28 534 (8.6%)	27 727 [97.2%]	807 [2.8%]
Western Australia	25 919 (7.8%)	23 886 [92.2%]	2033 [7.8%]
Australian Capital Territory	10 615 (3.2%)	10 426 [98.2%]	189 [1.8%]
Northern Territory	3547 (1.1%)	1718 [48.4%]	1829 [51.6%]
Tasmania	2604 (0.8%)	2584 [99.2%]	20 [0.8%]
Remoteness category (ARIA+)[2]			
Major city	196 706 (60.3%)	193 543 (61.4%)	3163 (28.6%)
Inner regional	79 206 (24.3%)	77 066 (24.5%)	2140 (19.3%)
Outer regional	40 544 (12.4%)	37 886 (12.0%)	2658 (24.0%)
Remote	5687 (1.7%)	4805 (1.5%)	882 (8.0%)
Very remote	4103 (1.3%)	1870 (0.6%)	2233 (20.2%)
Missing data	4466	4220	246
Socio-economic status (IRSAD),[3] median score (IQR)*	989 (943–1050)	991 (945–1051)	951 (897–991)
Hospital classification†			
Tertiary	128 409 (38.8%)	122 958 (38.5%)	5451 (48.2%)
Metropolitan	49 783 (15.1%)	47 926 (15.0%)	1857 (16.4%)
Regional/rural	43 891 (13.3%)	40 105 (12.6%)	3786 (33.4%)
Private	108 629 (32.9%)	108 401 (33.9%)	228 (2.0%)
Admission source†			
Operating theatre	184 308 (55.7%)	180 184 (56.4%)	4124 (36.4%)
Emergency department	85 896 (26.0%)	81 160 (25.4%)	4736 (41.8%)
Ward/cardiac care unit/high dependency unit	42 096 (12.7%)	40 677 (12.7%)	1419 (12.5%)
Other	18 390 (5.6%)	17 348 (5.4%)	1042 (9.0%)
Missing data	22	21	1

ARIA+ = Accessibility and Remoteness Index of Australia (2011); IRSAD = Index of Relative Socio-Economic Advantage and Disadvantage (2016); IQR = interquartile range.

* Missing data: 3681 patients (non-Indigenous, 3524 patients; Indigenous, 157 patients).

† As defined in Australian and New Zealand Intensive Care Society Centre for Outcome and Resource Evaluation Adult Patient Data base data dictionary.[4]

Table 3. Area-level Index of Relative Socio-economic Advantage and Disadvantage (IRSAD) of 327 031 people* admitted to intensive care in Australia, 2017–2019, by Indigenous status and decile

	Non-Indigenous patients		Indigenous patients		
IRSAD decile	Number	Proportion	Number	Proportion	Total
1	27094	8.6%	2952	26.4%	30046
2	29948	9.5%	1151	10.3%	31099
3	25007	7.9%	1161	10.4%	26168
4	35184	11.1%	1622	14.5%	36806
5	29195	9.2%	1167	10.4%	30362
6	30219	9.6%	801	7.2%	31020
7	27574	8.7%	540	4.8%	28114
8	32626	10.3%	716	6.4%	33342
9	38739	12.3%	713	6.4%	39452
10	40280	12.8%	342	3.1%	40622
Total	315866		11165		327031

* 315 866 non-Indigenous, 11 165 Indigenous patients for whom residential postcode could be mapped to IRSAD decile.

Table 4. Admission diagnosis categories for 330 712 people admitted to intensive care units (ICUs) in Australia, 2017–2019, by Indigenous status*

Admission diagnosis category	Non-Indigenous patients	Indigenous patients
Total number of patients	318 765	11 298
Cardiovascular	31 656 (9.9%)	1211 (10.7%)
Cardiac surgery	43 062 (13.5%)	1318 (11.7%)
Respiratory illness	46 979 (14.7%)	1877 (16.6%)
Neurology	38 524 (12.1%)	1053 (9.3%)
Gastrointestinal	55 284 (17.3%)	1271 (11.3%)
Sepsis	23 016 (7.2%)	1307 (11.6%)
Trauma	14 711 (4.6%)	858 (7.6%)
Other	65 533 (20.6%)	2403 (21.3%)
Missing data	625 (0.2%)	24 (0.2%)

* Data missing for 24 Indigenous and 625 non-Indigenous patients.

Table 5. Cox proportional hazards model (risk of death) and multivariable logistic regression (12-month mortality), each adjusted for Indigenous status, illness severity (APACHE III with age score removed), age (as a continuous covariate), socio-economic status, remoteness, hospital classification, region, and admission diagnosis

Characteristic	Patients Included	Deaths	HR (95% CI)	Adjusted HR (95% CI)	OR (95% CI)	Adjusted OR (95% CI)
Indigenous	11 322	1793 (15.8%)	1.01 (0.97–1.06)	1.20 (1.14–1.27)	1.01 (0.96–1.06)	1.24 (1.16–1.33)
APACHE III (less age score), per point	327 300*	51 781 (15.8%)	1.04 (1.04–1.04)	1.03 (1.03–1.03)	1.04 (1.04–1.04)	1.04 (1.04–1.04)
Age (per year)	327 300	51 781 (15.8%)	1.03 (1.03–1.03)	1.04 (1.03–1.04)	1.04 (1.04–1.04)1	1.04 (1.04–1.04)
IRSAD decile[3]						
1	30 046	5055 (9.8%)	1	1	1	1
2	31 099	5393 (10.5%)	1.04 (1.00–1.08)	1.06 (1.02–1.10)	1.04 (0.99–1.08)	1.04 (0.99–1.10)
3	26 168	4276 (8.3%)	0.97 (0.93 – 1.01)	1.00 (0.96–1.05)	0.97 (0.92–1.01)	1.01 (0.96–1.06)
4	36 806	6151 (12.0%)	0.99 (0.96–1.03)	1.06 (1.02–1.10)	0.99 (0.95–1.03)	1.03 (0.98–1.08)
5	30 362	4630 (9.0%)	0.90 (0.86 – 0.9)	1.03 (0.99–1.07)	0.89 (0.85–0.93)	1.00 (0.94–1.05)
6	31 020	4623 (9.0%)	0.88 (0.84–0.91)	1.02 (0.98–1.06)	0.87 (0.83–0.90)	0.99 (0.94–1.04)
7	28 114	4291 (8.4%)	0.90 (0.86–0.94)	1.00 (0.96–1.04)	0.89 (0.85–0.93)	0.99 (0.94–1.04)
8	33 342	4942 (9.6%)	0.87 (0.84–0.91)	1.00 (0.96–1.05)	0.86 (0.82–0.90)	0.98 (0.93–1.03)
9	39 452	5989 (11.7%)	0.89 (0.86–0.93)	1.02 (0.98–1.06)	0.88 (0.85–0.92)	0.99 (0.94–1.05)
10	40 622	6045 (11.8%)	0.87 (0.84–0.91)	1.00 (0.96–1.04)	0.86 (0.83–0.90)	1.00 (0.94–1.05)
Missing data	3681	506 (13.8%)	–	–	–	–
Remoteness (ARIA+)[2]						
Major city	196 706	31 244 (60.9%)	1	1	1	1
Inner regional	79 206	12 386 (24.1%)	0.98 (0.96–1.00)	1.02 (0.99–1.04)	0.98 (0.96–1.00)	0.99 (0.96–1.02)
Outer regional	40 544	6389 (12.5%)	0.99 (0.97–1.02)	1.02 (0.99–1.05)	0.99 (0.96–1.02)	0.99 (0.94–1.03)
Remote	5687	785 (1.5%)	0.86 (0.80–0.92)	0.96 (0.89–1.04)	0.85 (0.79–0.92)	0.96 (0.87–1.05)
Very remote	4103	504 (1.0%)	0.76 (0.69–0.83)	0.84 (0.76–0.92)	0.74 (0.68–0.81)	0.84 (0.74–0.96)
Missing data	4466	(593 (13.3%)	–	–	–	–
Hospital classification						
Tertiary	128 409	23 534 (18.3%)	1	1	1	1
Metropolitan	49 783	10 266 (20.6%)	0.96 (0.94–0.99)	0.94 (0.91–0.96)	1.16 (1.13–1.19)	1.03 (0.91–1.17)
Rural/regional	43 891	8356 (19.0%)	1.10 (1.07–1.13)	1.03 (1.00–1.06)	1.04 (1.02 – 1.08)	0.90 (0.79–1.02)
Private	108 629	9745 (9.0%)	0.44 (0.43–0.45)	0.69 (0.67–0.70)	0.44 (0.43–0.45)	0.66 (0.59–0.73)
Jurisdiction						
ACT	10 615	1456 (13.7%)	1	1	1	1
NSW	109 878	18 769 (17.1%)	1.26 (1.20–1.33)	1.08 (1.03–1.14)	1.30 (1.22–1.37)	1.44 (1.10–1.89)
NT	3547	479 (13.5%)	0.98 (0.88–1.09)	0.81 (0.72–0.91)	0.98 (0.89–1.09)	0.97 (0.62–1.53)
QLD	64 910	8705 (13.4%)	0.97 (0.92–1.02)	1.00 (0.94–1.05)	0.97 (0.92–1.03)	1.32 (1.01–1.74)
SA	28 534	4592 (16.1%)	1.19 (1.12 –1.26)	1.07 (0.01–1.14)	1.21 (1.13–1.29)	1.35 (0.99–1.83)

Characteristic	Patients Included	Deaths	HR (95% CI)	Adjusted HR (95% CI)	OR (95% CI)	Adjusted OR (95% CI)
TAS	2604	594 (22.8%)	1.75 (1.59–1.92)	1.35 (1.22–1.49)	1.86 (1.67–2.07)	1.93 (1.23–3.01)
VIC	84 705	13 611 (16.1%)	1.19 (1.13–1.25)	1.07 (1.01–1.13)	1.20 (1.14–1.28)	1.48 (1.13–1.94)
WA	25 919	3695 (14.3%)	1.04 (0.98–1.11)	1.07 (1.00–1.14)	1.05 (0.98–1.12)	1.39 (1.03–1.87)
Admission diagnosis†						
Cardiovascular	32 867	9034 (27.5%)	1	1	1	1
Respiratory	48 856	10 440 (21.4%)	0.72 (0.70–0.74)	1.22 (1.18–1.25)	0.72 (0.69–0.74)	1.34 (1.29–1.40)
Gastrointestinal	56 555	8176 (14.5%)	0.46 (0.45–0.48)	0.78 (0.75–0.80)	0.45 (0.43–0.46)	0.77 (0.74–0.80)
Neurological	39 577	6437 (16.3%)	0.53 (0.52–0.55)	1.14 (1.10–1.18)	0.51 (0.49–0.53)	1.23 (1.17–1.28)
Sepsis	24 323	7449 (30.6%)	1.10 (1.07–1.14)	0.98 (0.95–1.01)	1.16 (1.12–1.20)	0.99 (0.95–1.03)
Trauma	15 569	2015 (12.9%)	0.42 (0.40–0.44)	0.81 (0.78–0.86)	0.39 (0.37–0.41)	0.76 (0.71–0.80)
Cardiac surgery	44 380	1836 (4.1%)	0.13 (0.12–0.13)	0.23 (0.21–0.24)	0.11 (0.11–012)	0.20 (0.19–0.21)
Other	67 936	6414 (9.4%)	0.30 (0.29–0.30)	0.58 (0.56–0.60)	0.28 (0.27–0.28)	0.57 (0.55–0.60)
Missing data	649	100 (15.4%)	–	–	–	–

HR = hazard ratio; CI = confidence interval; OR = odds ratio; APACHE = Acute Physiology, Age and Chronic Health Evaluation; IRSAD = Index of Relative Socio-economic Advantage and Disadvantage;[3] ARIA = Accessibility/Remoteness Index of Australia.[2]

* Missing data (admission APACHE score not recorded): 299 (< 0.1%), including 49 people who died within twelve months of ICU admission.

† ANZICS-modified APACHE III categories.[4]

Table 6. Concordance between ANZICS Adult Patient Database (APD) and National Death Index (NDI) for inpatient death*

	NDI Status		
**APD Status**	**Alive**	**Died**	**Total**
Alive	405476	1198	406674
	99.71%	0.29%	100%
	99.76%	3.47%	92.22%
Died	982	33316	34298
	2.86%	97.14%	100%
	0.24%	96.53%	7.78%
Total	406458	34514	440972
	92.17%	7.83%	100%
	100%	100%	100%

First row has *frequencies*; second row has *row percentages* and third row has *column percentages.*
ANZICS – Australian and New Zealand Intensive Care Society
* Dataset used to assess concordance included episodes for which Indigenous status was missing from the APD database, as well as episodes with clear discordance between the APD and NDI (for example, death pre-dated ICU admission). This analysis is undertaken to assess the degree of agreement between the datasets for death as a means of context for the agreement between the datasets for Indigenous status.

Krippendorff's alpha with two variables = 0.97 (nominal method)

Table 7. Concordance between ANZICS Adult Patient Database (APD) and National Death Index (NDI) for Indigenous status of all people who died in hospital or within twelve months of discharge

	NDI Status		
**APD Status**	**Non-Indigenous**	**Indigenous**	**Total**
Non-Indigenous	66914	436	67350
	99.35%	0.65%	100%
	98.06%	27.97%	96.49%
Indigenous	1326	1123	2449
	54.14%	45.86%	100%
	1.94%	72.03%	3.51%
Total	68240	1559	69799
	97.77%	2.23%	100%
	100%	100%	100%

First row has *frequencies*; second row has *row percentages* and third row has *column percentages.*
ANZICS – Australian and New Zealand Intensive Care Society

Krippendorff's alpha with two variables = 0.55 (nominal method)

Table 8. Sensitivity analysis: selected demographic characteristics and outcomes after applying looser Indigenous status definition (recorded as Indigenous in either APD or NDI)

Characteristic	All patients	Non-Indigenous	Indigenous
Number of people	328 599	316 924	11 675
Age at admission (years), median [IQR]	66.0 [51.8 – 75.8]	66.5 [52.6 – 76.0]	51.6 [37.0- 63.9]
Sex			
Men	184 306 (56.1%)	178 289 (56.3%)	6017 (51.6%)
Women	144 018 (43.8%)	138 366 (43.7%)	5652 (48.4%)
Other	189 (<0.1%)	184 (0.1%)	5 (<0.1%)
Missing data	86 (<0.1%)	85 (<0.1%)	1 (<0.1%)
Planned admission†	154 006 (47.5%)	150 851 (48.3%)	3155 (27.1%)
Admission Diagnosis†			
Cardiovascular	31 989 (9.8%)	30 748 (9.7%)	1241 (10.7%)
Respiratory	48 601 (14.8%)	46 641 (14.8%)	1960 (16.8%)
Neurological	56 340 (17.2%)	54 999 (17.4%)	1341 (11.5%)
Sepsis	39 439 (12.0%)	38 331 (12.1%)	1108 (9.5%)
Trauma	23 874 (7.3%)	22 525 (7.1%)	1349 (11.6%)
Other	15 479 (4.7%)	14 612 (4.6%)	867 (7.4%)
Cardiac Surgery	67 920 (20.7%)	65 458 (20.7%)	2462 (21.1%)
APACHE III score; median (IQR)	46 [34–61]	46 [34–61]	46 [32–64]
Age score; median (IQR)	13 [5–17]	13 [5–17]	5 [0–11]
Acute physiology score; median (IQR)	33 [24–47]	33 [24–46]	38 [27–55]
ANZROD (%); median [IQR]	1.2 [0.4–5.2]	1.2 [0.4–5.2]	1.7 [0.5–6.7]
ICU length of stay (days); median [IQR]	1.7 [0.9–3.1]	1.7 [0.9–3.0]	1.9 [1.0–3.8]
Hospital length of stay (days); median [IQR]	7.8 [4.2–14.0]	7.8 [4.2–14.0]	7.3 [3.8–14.1]
Mechanical ventilation	104 552 (31.8%)	99 856 (31.5%)	4 696 (40.2%)
Primary outcome			
Time from ICU Admission to death (days)‡; median [IQR]	30 [6–145]	31 [6–146]	24 [5–131]
Unadjusted hazard ratio (95% CI)		1.08 (1.03–1.13)	
Secondary outcomes			
12-month mortality	49 734 (15.1%)	47 654 (15.0%)	2080 (17.8%)
Hospital mortality	22 154 (6.7%)	21 173 (6.7%)	981 (8.4%)
30-day mortality	24 925 (7.6%)	23 804 (7.5%)	1121 (9.6%)
90-day mortality	32 937(10.0%)	31 521 (10.0%)	1416 (12.1%)

APACHE = Acute Physiology and Chronic Health Evaluation; ANZROD = Australian and New Zealand Risk of Death.

† defined by ANZICS Data Dictionary[4]

† ANZICS-modified APACHE III categories[4]

‡ deceased patients only (49 734; non-Indigenous, 47 654; Indigenous, 2080)

All comparisons were significant p<0.001, with the exception of APACHE III score (p = 0.91), cardiac admission diagnosis (p = 0.81), other admission diagnosis (p = 0.13), hospital mortality (p = 0.07), and 30 day mortality (p = 0.12).

Table 9. Cox proportional hazards model (risk of death, aHR) and multivariable logistic regression (12-month mortality, aOR), each adjusted for Indigenous status, illness severity (APACHE III with age score removed), age (as a continuous covariate), socio-economic status, remoteness, hospital classification, region, and admission diagnosis, after applying looser Indigenous status definition (recorded as Indigenous in either APD or NDI)

Characteristic	Patients	Deaths	Hazard ratio	Adjusted HR	Odds ratio	Adjusted OR
Indigenous	11 675	2080 (18.8%)	1.21 (1.16–1.26)	1.45 (1.38–1.52)	1.22 (1.17–1.29)	1.55 (1.46–1.65)
APACHE III (less age score)	328 313	49 696 (15.1%)	1.03 (1.03–1.03)	1.03 (1.03–1.03)	1.04 (1.04–1.04)	1.04 (1.04–1.04)
Age	328 597	49 734 (15.1%)	1.03 (1.03–1.03)	1.04 (1.04–1.04)	1.04 (1.04–1.04)	1.04 (1.04–1.04)
IRSAD decile[3]						
1	29 881	4862 (16.3%	1	1	1	1
2	30 843	5150 (16.7%)	1.03 (0.99–1.07)	1.06 (1.02–1.10)	1.03 (0.99–1.08)	1.05 (1.00–1.10)
3	26 005	4119 (15.8%)	0.97 (0.93–1.01)	1.01 (0.97–1.06)	0.97 (0.93–1.01)	1.01 (0.96–1.07)
4	36 523	5868 (16.1%)	0.99 (0.95–1.02)	1.05 (1.01–1.10)	0.99 (0.94–1.03)	1.03 (0.98–1.09)
5	30 124	4418 (14.7%)	0.90 (0.86–0.93)	1.03 (0.98–1.07)	0.88 (0.85–0.92)	1.00 (0.95–1.05)
6	30 822	4424 (9.0%)	0.87 (0.84–0.91)	1.02 (0.98–1.06)	0.86 (0.83–0.90)	0.99 (0.93–1.04)
7	27 937	4112 (8.4%)	0.90 (0.86–0.93)	0.99 (0.95–1.04)	0.89 (0.85–0.93)	0.98 (0.93–1.04)
8	33 183	4739 (14.3%)	0.87 (0.83–0.90)	1.00 (0.96–1.04)	0.86 (0.82–0.90)	0.98 (0.92–1.03)
9	39 216	5754 (14.7%)	0.89 (0.86–0.93)	1.02 (0.98–1.06)	0.88 (0.85–0.92)	0.99 (0.94–1.04)
10	40 413	5820 (14.4%)	0.87 (0.84–0.91)	0.99 (0.95–1.03)	0.87 (0.83–0.90)	0.98 (0.93–1.04)
Remoteness (ARIA+)[2]						
Major city	195 459	29 986 (15.3%)	1	1	1	1
Inner regional	78 658	11 818 (15.0%)	0.98 (0.96–1.00)	1.02 (1.00–1.05)	0.98 (0.95–1.00)	0.99 (0.96–1.02)
Outer regional	40 301	6144 (15.3%)	0.99 (0.97–1.02)	1.02 (0.99–1.05)	0.99 (0.96–1.02)	0.98 (0.94–1.03)
Remote	5669	746 (13.2%)	0.85 (0.79–0.91)	0.95 (0.88–1.02)	0.84 (0.77–0.90)	0.93 0.84–1.02)
Very Remote	4081	489 (12.0%)	0.77 (0.70–0.84)	0.77 (0.70–0.86)	0.75 (0.68–0.83)	0.78 (0.69–0.89)
Hospital classification						
Tertiary	127 358	22 470 (17.6%)	1	1	1	1
Metropolitan	49 194	9720 (19.8%)	0.96 (0.94–0.99)	0.93 (0.91–0.95)	1.15 (1.12–1.18)	1.01 (0.89–1.14)
Rural	43 492	7958 (18.3%)	1.10 (1.06–1.13)	1.01 (0.98–1.04)	1.05 (1.02–1.08)	0.89 (0.78–1.01)
Private	108 555	9586 (8.8%))	0.45 (0.4–0.47)	0.69 (0.67–0.71)	0.45 (0.44–0.46)	0.66 (0.59–0.74)
Jurisdiction of ICU admission						
Australian Capital Territory	10 546	1396 (13.2%)	1	1	1	1
New South Wales	109 179	18 025 (16.5%)	1.26 (1.20–1.34)	1.08 (1.03–1.15)	1.30 (1.22–1.37)	1.47 (1.13–1.92)
Northern Territory	3522	465 (13.2%)	0.99 (0.89–1.10)	0.78 (0.70–0.88)	1.00 (0.89–1.12)	0.96 (0.61–1.49)
Queensland	64 563	8357 (12.9%)	0.97 (0.92–1.03)	1.00 (0.94–1.06)	0.97 (0.92–1.04)	1.34 (1.02–1.75)
South Australia	28 322	4360 (15.4%)	1.18 (1.11–1.25)	1.06 (1.00–1.13)	1.20 (1.12–1.27)	1.36 (1.01–1.84)
Tasmania	2579	558 (21.6%)	1.72 (1.55–1.89)	1.34 (1.21–1.49)	1.81 (1.62–2.02)	1.93 (1.24–2.98)
Victoria	84 108	13 035 (15.5%)	1.19 (1.12–1.25)	1.08 (1.02–1.14)	1.20 (1.13–1.28)	1.50 (1.15–1.96)
Western Australia	25 780	3538 (13.7%)	1.04 (0.98–1.11)	1.05 (0.99–1.12)	1.04 (0.98–1.11)	1.39 (1.03–1.86)

Characteristic	Patients	Deaths	Hazard ratio	Adjusted HR	Odds ratio	Adjusted OR
Admission diagnosis†						
Cardiovascular	31 989	8171 (25.5%)	1	1	1	1
Respiratory	48 601	10 182 (21.0%)	0.77 (0.75–0.79)	1.28 (1.24–1.32)	0.77 (0.75–0.80)	1.40 (1.35–1.45)
Gastrointestinal	56 340	7935 (14.1%)	0.50 (0.48–0.51)	0.81 (0.78–0.84)	0.48 (0.46–0.49)	0.80 (0.77–0.84)
Neurological	39 439	6285 (15.9%)	0.57 (0.55–0.59)	1.19 (1.15–1.24)	0.55 (0.53–0.57)	1.28 (1.22–1.33)
Sepsis	23 874	7054 (29.6%)	1.16 (1.12–1.20)	1.01 (0.98–1.04)	1.22 (1.18–1.27)	1.02 (0.98–1.07)
Trauma	15 479	1933 (12.5%)	0.44 (0.42–0.47)	0.84 (0.80–0.88)	0.41 (0.39–0.44)	0.78 (0.73–0.83)
Other	67 920	6301 (9.3%)	0.32 (0.31–0.33)	0.61 (0.59–0.63)	0.30 (0.29–0.31)	0.60 (0.58–0.68)
Cardiac surgery	44 300	1782 (4.0%)	0.14 (0.13–0.14)	0.23 (0.22–0.24)	0.12 (0.12–0.13)	0.20 (0.19–0.22)

APACHE III = Acute Physiology and Chronic Health Evaluation III; IQR = interquartile range; IRSAD – Index Relative Socio-economic Advantage and Disadvantage[1];ARIA = Accessibility/Remoteness Index of Australia.[2]
† ANZICS Modified APACHE III categories.[4]

Table 10. Comparison of characteristics of intensive care unit episodes with and without SLK581 data linkage keys

Characteristic	All patients	Non-linked episodes‡	Linked episodes
Number of people	374 594	43 882	330 712
Age at admission (years), median (IQR)	65.8 [51.5–75.6]	63.7 [51.2–75.8]	66.1 [51.9–75.8]
Sex			
Men	210 399 (56.2%)	24 879 (56.7%)	185 520 (56.1%)
Women	163 917 (43.8)	19 000 (43.3%)	144 917 (43.8%)
Other	189 (0.11%)	0	189 (0.1%)
Missing data	89 (<0.1%)	3 (<0.1%)	86 (<0.1%)
State (ICU admission)			
New South Wales	125 622 (33.5%)	15 738 [35.9%]	109 884 [33.2%]
Victoria	96 443 (25.8%)	11 750 [26.8%]	84 689 [25.6%]
Queensland	73 449 (19.6%)	8543 [19.5%]	64 906 [19.6%]
South Australia	31 689 (8.5%)	3163 [7.2%]	28 526 [8.6%]
Western Australia	28 342 (7.6%)	2416 [5.5%]	25 926 [7.8%]
Australian Capital Territory	10 774 (2.9%)	164 [0.4%]	10 610 [3.2%]
Northern Territory	4028 (1.1%)	464 [1.1%]	3564 [1.1%]
Tasmania	4247 (1.1%)	1640 [3.7%]	2607 [0.8%]
Hospital classification†			
Tertiary	148 430 (39.6%)	20 037 (45.7%)	128 393 (38.8%)
Metropolitan	56 774 (15.2%)	7066 (16.%)	49 708 (15.1%)
Regional/rural	51 424 (13.7%)	7461 (17.0%)	43 963 (13.3%)
Private	117 966 (31.5%)	9318 (21.2%)	108 648 (32.9%)
Admission source†			
Operating theatre	207 402 (55.4%)	23 172 (52.9%)	184 230 (55.7%)
Emergency department	98 712 (26.4%)	12 691 (29.0%)	86 021 (26.0%)
Ward/cardiac care unit/high dependency unit	47 286 (12.6%)	5293 (12.1%)	41 993 (12.7%)
Other	21 125 (5.6%)	2680 (6.1%)	18 445 (5.6%)
ICU†	116 764 (31.2%)	11 559 (26.3%)	105 205 (31.8%)
APACHE III, median score (IQR)	46 (34–61)	47 (34–61)	46 (34–62)
Risk of death, median (IQR)	4.3% (1.6–12.8)	4.5% (1.7–12.1)	4.3% (1.6–13.0)
ANZROD, median (IQR)	1.3% (0.4–5.3)	1.4% (0.5–4.7)	1.3% (0.4–5.4)
Mechanical Ventilation	120 410 (32.1%)	14 668 (33.4%)	105 742 (32.0%)

All comparisons are significant ($p < 0.001$)

ANZROD = Australia and New Zealand Risk of Death; APACHE III = Acute Physiology and Chronic Health Evaluation III; IQR = interquartile range.

* As defined by the Australian and New Zealand Intensive Care Society Centre for Outcome and Resource Evaluation Adult Patient Database (ANZICS CORE APD) data dictionary.[4]

† As defined by the ANZICS CORE APD data dictionary: admissions of patients requiring one of the following: "invasive ventilation; non-invasive ventilation (> 50% of stay or continuously > 6 h); 1:1 nursing; continuous renal replacement therapy." Admissions of patients requiring the specific expertise of the ICU environment but not fitting this definition are defined as high dependency unit admissions.[4]

‡ Critically ill patients not allocated a statistical linkage key (SLK581).

Table 11. Contributing sites

Albury Wodonga Health ICU	Central Gippsland Health Service (Sale)	Hurstville Private Hospital ICU
Alfred Hospital ICU	Coffs Harbour Health Campus ICU	Ipswich Hospital ICU
Alice Springs Hospital ICU	Concord Hospital (Sydney) ICU	John Fawkner Hospital ICU
Angliss Hospital ICU	Dandenong Hospital ICU	John Flynn Private Hospital ICU
Armadale Health Service ICU	Dubbo Base Hospital ICU	John Hunter Hospital ICU
Ashford Community Hospital ICU	Epworth Eastern Private Hospital ICU	Joondalup Health Campus ICU
Austin Hospital ICU	Epworth Freemasons Hospital ICU	Kareena Private Hospital ICU
Ballarat Health Services ICU	Epworth Geelong ICU	Knox Private Hospital ICU
Bankstown-Lidcombe Hospital ICU	Epworth Hospital (Richmond) ICU	Latrobe Regional Hospital ICU
Bathurst Base Hospital ICU	Fairfield Hospital ICU	Launceston General Hospital ICU
Bendigo Health Care Group ICU	Fiona Stanley Hospital ICU	Lingard Private Hospital ICU
Blacktown Hospital ICU	Flinders Medical Centre ICU	Lismore Base Hospital ICU
Box Hill Hospital ICU	Flinders Private Hospital ICU	Liverpool Hospital ICU
Brisbane Private Hospital ICU	Footscray Hospital ICU	Logan Hospital ICU
Buderim Private Hospital ICU	Frankston Hospital ICU	Lyell McEwin Hospital ICU
Bunbury Regional Hospital ICU	Gold Coast Private Hospital ICU	Mackay Base Hospital ICU
Bundaberg Base Hospital ICU	Gold Coast University Hospital ICU	Macquarie University Private Hospital
Caboolture Hospital ICU	Gosford Hospital ICU	Maitland Hospital ICU
Cabrini Hospital ICU	Gosford Private Hospital ICU	Maitland Private Hospital ICU
Cairns Hospital ICU	Goulburn Base Hospital ICU	Manly Hospital & Community Health ICU
Calvary Adelaide Hospital ICU	Goulburn Valley Health ICU	Manning Rural Referral Hospital ICU
Calvary Hospital (Canberra) ICU	Grafton Base Hospital ICU	Maroondah Hospital ICU
Calvary John James Hospital ICU	Greenslopes Private Hospital ICU	Mater Adults Hospital (Brisbane) ICU
Calvary Mater Newcastle ICU	Griffith Base Hospital ICU	Mater Health Services North Queensland
Calvary North Adelaide Hospital ICU	Hervey Bay Hospital ICU	Mater Private Hospital (Brisbane) ICU
Campbelltown Hospital ICU	Hollywood Private Hospital ICU	Mater Private Hospital (Sydney) ICU
Canberra Hospital ICU	Holmesglen Private Hospital ICU	Sunshine Coast University Hospital ICU
Melbourne Private Hospital ICU	Royal Brisbane and Women's Hospital	Sunshine Coast University Private
Mildura Base Public Hospital ICU	Royal Darwin Hospital ICU	Sunshine Hospital ICU
Monash Medical Centre-Clayton Campus	Royal Melbourne Hospital ICU	Sutherland Hospital & Community
Mount Hospital ICU	Royal North Shore Hospital ICU	Sydney Adventist Hospital ICU
Mount Isa Hospital ICU	Royal Perth Hospital ICU	Sydney Southwest Private Hospital ICU
Mulgrave Private Hospital ICU	Royal Prince Alfred Hospital ICU	Tamworth Base Hospital ICU
National Capital Private Hospital ICU	Shoalhaven Hospital ICU	The Bays Hospital ICU
Nepean Hospital ICU	Sir Charles Gairdner Hospital ICU	The Chris O'Brien Lifehouse ICU
Newcastle Private Hospital ICU	South West Healthcare (Warrnambool)	The Memorial Hospital (Adelaide) ICU
Noosa Hospital ICU	St Andrew's Hospital (Adelaide) ICU	The Northern Hospital ICU
North Shore Private Hospital ICU	St Andrew's Hospital Toowoomba ICU	The Prince Charles Hospital ICU
North West Regional Hospital (Burnie)	St Andrew's Private Hospital (Ipswich)	The Queen Elizabeth (Adelaide) ICU
Northeast Health Wangaratta ICU	St Andrew's War Memorial Hospital ICU	The Wesley Hospital ICU
Northern Beaches Hospital ICU	St George Hospital (Sydney) ICU	Toowoomba Hospital ICU
Norwest Private Hospital ICU	St George Private Hospital (Sydney) ICU	Townsville University Hospital ICU
Orange Base Hospital ICU	St John of God (Berwick) ICU	Tweed Heads District Hospital ICU
Peninsula Private Hospital ICU	St John Of God Health Care (Subiaco) ICU	University Hospital Geelong ICU
Pindara Private Hospital ICU	St John of God Hospital (Bendigo) ICU	Wagga Wagga Base Hospital & District
Port Macquarie Base Hospital ICU	St John Of God Hospital (Geelong) ICU	Warringal Private Hospital ICU
Prince of Wales Hospital (Sydney) ICU	St John Of God Hospital (Murdoch) ICU	Werribee Mercy Hospital ICU
Prince of Wales Private Hospital (Sydney)	St John of God Midland Public & Private	Western District Health Service
Princess Alexandra Hospital ICU	St Vincent's Private Hospital Northside	Western Hospital (SA) ICU
Queen Elizabeth II Jubilee Hospital ICU	St Vincent's Hospital (Melbourne) ICU	Western Private Hospital ICU
Redcliffe Hospital ICU	St Vincent's Hospital (Sydney) ICU	Westmead Hospital ICU
Robina Hospital ICU	St Vincent's Hospital (Toowoomba) ICU	Westmead Private Hospital ICU
Rockhampton Hospital ICU	St Vincent's Private Hospital (Sydney)	Wimmera Health Care Group (Horsham)
Rockingham General Hospital ICU	St Vincent's Private Hospital Fitzroy ICU	Wollongong Hospital ICU
Royal Adelaide Hospital ICU	Sunnybank Hospital ICU	Women's and Children's Hospital PICU
Wollongong Private Hospital ICU	Hornsby Ku-ring-gai Hospital ICU	Wyong Hospital ICU

Data sharing statement

What data will be shared?
Individual participant data that underlie the results reported in this article, after de-identification (text, tables, figures and appendices)

When will data be available?
Beginning 9 months and ending 60 months following article publication.

With whom?
Researchers who provide a methodologically sound proposal, and whose proposed use of the data has been approved by an independent human research ethics committee.

For what types of analyses?
To achieve aims in the approved proposal.

By what mechanism will data be made available?
Proposals may be submitted up to 60 months following publication, and proposals should be directed to the corresponding author. To gain access, data requesters will be required to signed a data access agreement.

References

1. von Elm E, Altman DG, Egger M, et al; STROBE Initiative. The Strengthening the Reporting of Observational Studies in Epidemiology (STROBE) statement: guidelines for reporting observational studies. Lancet 2007; 370: 1453-1457.
2. Hugo Centre for Migration and Population Research (University of Adelaide). Accessibility/remoteness index of Australia (ARIA) 2011. Updated 30 Nov 2021. https://able.adelaide.edu.au/hugo-centre/services/aria (viewed Aug 2022).
3. Australian Bureau of Statistics. Socio-Economic Indexes for Areas (SEIFA) 2011 (2033.0.55.001) [technical paper]. 28 Mar 2013. http://www.ausstats.abs.gov.au/ausstats/subscriber.nsf/LookupAttach/2033.0.55.001Publication28.03.131/$File/2033.0.55.001%20SEIFA%202011%20Technical%20Paper.pdf (viewed Nov 2019).
4. Australian and New Zealand Intensive Care Society (ANZICS) Centre for Outcomes and Resource Evaluation (CORE). APD data dictionary. ANZICS CORE: adult patient database, version 6.1. Apr 2022. https://www.anzics.com.au/wp-content/uploads/2021/03/ANZICS-APD-Data-Dictionary.pdf (viewed Aug 2022).
